# Development and Validation of *Burkholderia pseudomallei*-Specific Real-Time PCR Assays for Clinical, Environmental or Forensic Detection Applications

**DOI:** 10.1371/journal.pone.0037723

**Published:** 2012-05-18

**Authors:** Erin P. Price, Julia L. Dale, James M. Cook, Derek S. Sarovich, Meagan L. Seymour, Jennifer L. Ginther, Emily L. Kaufman, Stephen M. Beckstrom-Sternberg, Mark Mayo, Mirjam Kaestli, Mindy B. Glass, Jay E. Gee, Vanaporn Wuthiekanun, Jeffrey M. Warner, Anthony Baker, Jeffrey T. Foster, Patrick Tan, Apichai Tuanyok, Direk Limmathurotsakul, Sharon J. Peacock, Bart J. Currie, David M. Wagner, Paul Keim, Talima Pearson

**Affiliations:** 1 Center for Microbial Genetics and Genomics, Northern Arizona University, Flagstaff, Arizona, United States of America; 2 Menzies School of Health Research, Casuarina, Northern Territory, Australia; 3 Translational Genomics Research Institute, Phoenix, Arizona, United States of America; 4 Centers for Disease Control and Prevention, Atlanta, Georgia, United States of America; 5 Mahidol-Oxford Tropical Medicine Research Unit, Faculty of Tropical Medicine, Mahidol University, Bangkok, Thailand; 6 Microbiology and Immunology, School of Veterinary and Biomedical Sciences, James Cook University, Townsville, Queensland, Australia; 7 Genome Institute of Singapore, Singapore, Singapore; 8 Department of Microbiology and Immunology, Faculty of Tropical Medicine, Mahidol University, Bangkok, Thailand; 9 Department of Medicine, University of Cambridge, Cambridge, United Kingdom; Columbia University, United States of America

## Abstract

The bacterium *Burkholderia pseudomallei* causes melioidosis, a rare but serious illness that can be fatal if untreated or misdiagnosed. Species-specific PCR assays provide a technically simple method for differentiating *B. pseudomallei* from near-neighbor species. However, substantial genetic diversity and high levels of recombination within this species reduce the likelihood that molecular signatures will differentiate all *B. pseudomallei* from other Burkholderiaceae. Currently available molecular assays for *B. pseudomallei* detection lack rigorous validation across large *in silico* datasets and isolate collections to test for specificity, and none have been subjected to stringent quality control criteria (accuracy, precision, selectivity, limit of quantitation (LoQ), limit of detection (LoD), linearity, ruggedness and robustness) to determine their suitability for environmental, clinical or forensic investigations. In this study, we developed two novel *B. pseudomallei* specific assays, 122018 and 266152, using a dual-probe approach to differentiate *B. pseudomallei* from *B. thailandensis*, *B. oklahomensis* and *B. thailandensis*-like species; other species failed to amplify. Species specificity was validated across a large DNA panel (>2,300 samples) comprising *Burkholderia* spp. and non*-Burkholderia* bacterial and fungal species of clinical and environmental relevance. Comparison of assay specificity to two previously published *B. pseudomallei*-specific assays, BurkDiff and TTS1, demonstrated comparable performance of all assays, providing between 99.7 and 100% specificity against our isolate panel. Last, we subjected 122018 and 266152 to rigorous quality control analyses, thus providing quantitative limits of assay performance. Using *B. pseudomallei* as a model, our study provides a framework for comprehensive quantitative validation of molecular assays and provides additional, highly validated *B. pseudomallei* assays for the scientific research community.

## Introduction

The *Burkholderia* genus contains over 60 species, some of which are of environmental, clinical or forensic importance. With the exception of the obligate mammalian pathogen, *B. mallei*, the *Burkholderia* spp. reside in many different environmental niches that include fresh and salt water, soil, and the plant rhizosphere [1], [2]. Certain *Burkholderia* spp. including *B. ambifaria*, *B. anthina*, *B. cenocepacia*, *B. cepacia*, *B. dolosa*, *B. mallei*, *B. multivorans*, *B. oklahomensis*, *B. pseudomallei*, *B. pyrrocinia*, *B. stabilis*, *B. thailandensis*, *B. ubonensis* and *B. vietnamiensis* have been shown to cause opportunistic infections in humans [1], [2], [3], [4], [5]. Of these species, *B. pseudomallei* is of greatest clinical relevance, being the most common cause of fatal community-acquired bacteremia in northeast Thailand [6] and fatal community-acquired bacteremic pneumonia in Northern Australia [7]. *B. pseudomallei* and *B. mallei* are important from a forensic standpoint due to the disease severity caused by these species and their bioweaponization potential, with both species listed as Category B Select Agents by the Centers for Disease Control and Prevention (http://www.bt.cdc.gov/agent/agentlist-category.asp).

*B. pseudomallei* may not be readily identifiable from clinical, forensic or environmental samples based on culturing alone, as multiple morphotypes exist for this species, even within the same strain [8], [9]. Further, many *Burkholderia* spp. co-reside with *B. pseudomallei* in the environment and can appear morphologically and serologically similar to *B. pseudomallei*, even when using selective culture media, or biochemical and serological tests designed to solely detect *B. pseudomallei* [10], [11]. Latex agglutination methods are routinely used in endemic areas such as Thailand and northern Australia and have shown good, but not perfect, specificity for *B. pseudomallei* [12]. Accurate identification of *B. pseudomallei* is particularly difficult in non-endemic regions where selective media are typically not used to isolate *B. pseudomallei* and technicians lack the experience required to identify putative *B. pseudomallei* isolates. Therefore, positive *B. pseudomallei* identification cannot be based solely on phenotypic characteristics and molecular characterization is a necessary component of definitive species assignment [13].

Two striking features of *B. pseudomallei* are its genetic and genomic heterogeneity [14], [15], [16], [17] and high rates of recombination [18]. These factors render accurate *B. pseudomallei* identification using molecular methods a non-trivial endeavor. A number of *B. pseudomallei-*specific molecular signatures have been described in the literature [13], [19], [20], [21], [22], [23], [24], [25], [26]. The vast majority of these signatures, however, have been identified using limited *in silico* comparative genomic data; the likelihood of false-positive (i.e. shared with neighboring species) and false-negative (i.e. not universally found within the target species) signatures is therefore reasonably high. Compounding this issue, few signatures have been tested against *Burkholderia* and non-*Burkholderia* spp. panels that adequately sample existing genetic diversity and, therefore, more accurately validate specificity. Indeed, one promising species-specific *B. pseudomallei* signature [24] gave multiple false-positive results following screening across a more diverse species panel [27]. It is thus difficult to develop 100% accurate *B. pseudomallei*-specific assays despite the importance of this bacterium from a clinical, environmental and forensic stance.

The current ‘gold standard’ species-specific assay for *B. pseudomallei* relies on amplification of *orf2* of the type three secretion system 1 (TTS1) cluster, which is only present in *B. pseudomallei* [13]. More recently, the BurkDiff assay was developed as a dual-probe TaqMan assay to differentiate *B. pseudomallei* from *B. mallei* [27]. Both TTS1 and BurkDiff have been tested against *Burkholderia* and non-*Burkholderia* spp. strain panels of moderate size and have shown promising speciation accuracy. However, although the TTS1 and BurkDiff assays appear to be highly reliable for identification of *B. pseudomallei* [13], [23], [26], [27], [28], [29], both assays give null results for other *Burkholderia* spp. that can phenotypically resemble *B. pseudomallei*, such as *B. thailandensis*, *B. thailandensis-*like species [30], *B. oklahomensis*, *B. vietnamiensis* or *B. ubonensis* [11], [31], [32], [33], meaning that these other *Burkholderia* species often go unidentified and thus their true incidence is largely unknown. In addition, neither assay has been comprehensively validated against a wide range of rigorous performance criteria [34], although both assays have demonstrated an impressive limit of detection [13], [27], and for TTS1, high selectivity in complex clinical and environmental specimens [13], [26], [35].

Based on these existing knowledge gaps, the high predicted likelihood that any *B. pseudomallei* specific assay will sometimes produce false results and the importance of robust detection assays for clinical, environmental and forensic purposes, our aims were as follows. First, to identify *B. pseudomallei*-specific single nucleotide polymorphisms (SNPs) using whole genome sequence (WGS) data, with a view to providing additional speciation markers that enable differentiation of *B. pseudomallei* from *B. mallei*, *B. thailandensis*, *B. oklahomensis* and *B. thailandensis*-like species. Second, to develop real-time PCR assays for these targets using the robust dual-probe TaqMan [36] format. Third, to screen our TaqMan *B. pseudomallei* assays, and the TTS1 and BurkDiff assays, across an extensive panel of 2,332 *Burkholderia* spp. and non-*Burkholderia* DNA to determine specificity. Last, to quantitatively assess the accuracy, specificity, precision, selectivity, limit of quantitation (LoQ), limit of detection (LoD), linearity, ruggedness and robustness of our TaqMan assays by pushing them to their performance limits, which provides important information on assay performance for downstream applications.

## Materials and Methods

### Bacterial growth conditions and DNA preparation

All *Burkholderia* spp., with the exception of *B. mallei*, were cultured on Luria Bertani (LB) agar (Becton Dickinson, Franklin Lakes NJ); *B. mallei* LB plates were further supplemented with 4% vol/vol glycerol (Thermo Fisher Scientific, Pittsburgh PA) [37]. *Burkholderia* DNA extractions were performed from pure cultures as previously described [38]. For non-*Burkholderia* bacterial species, cultures were grown using appropriate agar and atmospheric conditions (Hardy Diagnostics, Santa Maria, CA; Becton Dickinson) and extracted using either the Gram-positive or Gram-negative protocols of the DNeasy Blood and Tissue kit (Qiagen, Valencia CA), as appropriate. For *Staphylococcus* and *Streptococcus* species, lysostaphin or mutanolysin (Sigma-Aldrich, St Louis, MO) was added to the DNeasy lysis buffer, respectively, to improve extraction efficiency. For yeast and fungal species, we used the DNeasy Blood and Tissue kit (Qiagen) as per manufacturer's instructions for yeast extraction. All DNA samples were quantified using a NanoDrop 8000 spectrophotometer (Thermo Fisher Scientific) and normalized to either 1 or 2 ng/µL in 1× TE (pH 8.0; Thermo Fisher Scientific) for direct use in PCR.

### SNP discovery

*B. pseudomallei*-specific SNP signatures were identified using a two-pronged approach. First, we used an in-house pipeline [18] to identify orthologous SNP loci among 35 *Burkholderia* genomes (24 *B. pseudomallei* (1026b, 1106a, 1106b, 112, NCTC 13177, 14, MSHR1655, 1710a, 1710b, 22, MSHR305, 406e, 576, 668, 7894, 9, 91, B7210, BCC215, DM98, E208, K96243, Pasteur 52237 and S13), six *B. thailandensis* (381, 700388, Bt4, TXDOH, E254 and E264), two *B. oklahomensis* (C6786 and EO147), and one each of *B. dolosa* (AUO158), *B. ubonensis* (Bu) and *B. thailandensis*-like MSMB 43 [30]. BLAST [39] analysis was performed on candidate SNP loci to identify SNPs in genetic regions absent in *B. mallei*. This additional filter was performed because the *B. pseudomallei* clade includes *B. mallei* [40]. As a result, many evolutionarily stable SNP alleles shared among all *B. pseudomallei* will also be shared with *B. mallei* and, therefore, will not be *B. pseudomallei*-specific. Second, we validated species specificity by comparing potential *B. pseudomallei*-specific signatures *in silico* against all available *B. pseudomallei*, *B. mallei*, *B. thailandensis*, *B. thailandensis-*like, *B. vietnamiensis*, *B. oklahomensis*, *B. ubonensis* and *B. cepacia* genomes (as of September 2010). Of five shortlisted signatures (chosen for the conserved nature of their surrounding sequence), only two (122018 and 266152) were investigated further as the others lacked specificity for *B. pseudomallei* upon BLAST analysis across these other *Burkholderia* spp.

### Assay design and PCR conditions

The *B. pseudomallei*-specific SNPs 122018 and 266152 (arbitrarily named) are bi-allelic, with *B. pseudomallei* containing one SNP state and *B. thailandensis*, *B. oklahomensis* and *B. thailandensis-*like species containing the alternate state. Other Burkholderiaceae possess additional SNPs or indels that would adversely affect binding of the *B. pseudomallei*-specific probe according to *in silico* analysis. Once *in silico B. pseudomallei* specificity was determined, the SNP signatures were converted to TaqMan MGB probe format [36]. TaqMan probes and primers (Table 1) were designed using Primer Express v3.0 software (Applied Biosystems, Foster City CA). All primers and probes were subject to BLAST analysis to confirm specificity. PCRs were performed in 384-well optical plates using 1× TaqMan Universal PCR Master Mix (Applied Biosystems), primers and probes, and molecular-grade H_2_O (Invitrogen). One µL DNA template (equating to 2 ng, or 2.5×10^5^ genomic equivalents) was added per reaction to a final volume of 10 µL. All reactions were carried out in dual-probe format and in duplicate using 2 ng DNA template (1 ng template was used for specificity screening), unless otherwise specified. For the 122018 assay, primer and probe concentrations were 0.3 µM and 0.1 µM, respectively, whereas the 266152 assay used concentrations at 0.3 µM and 0.2 µM, respectively. Thermocycling was conducted using default conditions (2 min at 50°C, 10 min at 95°C followed by 40 cycles of denaturation for 15 s at 95°C and annealing and extension for 1 min at 60°C) on a 7900HT Real-Time PCR System (Applied Biosystems).

**Table 1:** *B. pseudomallei* 122018 and 266152 assays designed in this study.

Assay	Primers and probes (5′-3′)^a^		SNP location^b^	Expected polymorphism^c^
122018				
		CCTGATCGCCCGTCTTCG	3,713,843 (Chr1)	T = *B. pseudomallei*
		CGCAAAACTTTCTGGGGTAGT		C = other species
		6FAM-CCAGCGATTTGTTGAA		
		VIC-CAACGACTTGTTGAAC		
266152				
		aataaatcataaACGTGAGGCCGGAGATGT	846,056 (Chr2)	T = *B. pseudomallei*
		aataaatcataaGACCGACATCACGCACAGC		C = other species
		VIC-CGGTCTACACGCATGA		
		6FAM-CGGTCTACACGCACGA		

a Underlined nucleotides indicate the position of the SNP in the TaqMan probe; lowercase nucleotides indicate a deliberately incorporated 5′ flap to enhance amplification efficiency [43].

b Based on *B. pseudomallei* K96243 genome (GenBank Accession numbers BX571965 and BX571966 for chromosomes 1 and 2, respectively) [14].

c ‘Other species’ refers specifically to *B. thailandensis*, *B. oklahomensis* and *B. thailandensis-*like spp. *B. mallei* and other *Burkholderia* spp. (e.g. *B. vietnamiensis*, *B. ubonensis* etc.) do not amplify according to *in silico* and wet-bench analyses.

### Previously established *B. pseudomallei*-specific assays

Genotyping calls for the 122018 and 266152 assays were compared with two established *B. pseudomallei*-specific real-time PCR assays, BurkDiff [27] and TTS1 [13], to determine the specificity of all four assays (see ‘Assay quality performance’ below for details). BurkDiff is a dual-probe TaqMan assay that differentiates *B. pseudomallei* and *B. mallei*; other *Burkholderia* spp. fail to amplify due to high levels of sequence diversity. We made a modification to the BurkDiff assay (0.2 µM each primer/reaction rather than the 0.9 µM previously reported) to improve assay efficiency. TTS1 is a single probe assay that detects the presence or absence of *B. pseudomallei*; the gene targeted by this assay is absent in other *Burkholderia* spp. [13]. We performed TTS1 detection essentially as described elsewhere [41] but with the following alterations: we maintained the originally described primer and probe concentrations [13], and used default thermocycling parameters on the AB7900HT instrument for consistency with the 122018 and 266152 assays.

### Assay quality performance

To determine the suitability of our new *B. pseudomallei*-specific assays over a wide range of conditions, we tested the performance of the 122018 and 266152 assays across several criteria; accuracy, specificity, precision, selectivity, LoQ, LoD, linearity, ruggedness and robustness (Methods S1). We designed quality performance experiments based on standardized definitions of these parameters [34]. Two representative samples, *B. pseudomallei* 104 and *B. thailandensis-*like MSMB 43, were used to test parameters due to inherent differences in probe efficiencies between these different species. Species specificity for the 122018 and 266152 assays was determined by screening them across our entire *Burkholderia* DNA collection, which comprises 2,205 *Burkholderia* spp. samples (Table 2), normalized to 1 ng/µL using the NanoDrop 8000 instrument. We also tested these assays across 127 common soil, water or clinically important prokaryotic and eukaryotic species to confirm specificity (Table S2). A 466 bp real-time SYBR Green 16 S PCR [42] was used to confirm DNA integrity in instances where no amplification with the *B. pseudomallei* assays was observed, including fungal and yeast species, which amplified (albeit less robustly, but above NTC signal) with this primer set, probably due to non-specific amplification of other rRNA regions. NTCs were included as cycles-to-threshold controls due to delayed but positive amplification using AmpliTaq Gold polymerase, which contains endogenous *E. coli* 16 S RNA.

**Table 2:** *Burkholderia* spp. DNA specificity panel used in this study.

*Burkholderia* species^a^	No. samples
*B. pseudomallei*	1,954
*B. thailandensis*	86
*B. mallei*	76
*B. ubonensis*	32
*B. thailandensis-*like/*B. thailandensis*/*B. oklahomensis* b	28
*B.* spp.^c^	13
*B. cepacia*	6
*B. oklahomensis*	4
*B. vietnamiensis*	2
*B. thailandensis-*like	2
*B. cenocepacia*	1
*B. phytofirmans*	1
**Total**	**2,205**

a According to genotyping results generated in the current study, 16 S sequencing or MLST.

b Previously identified as ‘*Burkholderia* spp.’ and renamed *Burkholderia thailandensis/B. thailandensis-*like*/B. oklahomensis* based on their genotyping outcomes in this study. However, we could not accurately differentiate these three species as they all amplify the non-*B. pseudomallei* probe in the 122018 and 266152 assays. Neither TTS1 nor BurkDiff assays amplify *B. thailandensis*, *B. thailandensis-*like or *B. oklahomensis* species, and thus could not be used for species assignment.

c Ruled out as being *B. pseudomallei*, *B. thailandensis*, *B. thailandensis-*like, *B. oklahomensis*, *B. vietnamiensis* and *B. ubonensis* but strongly suspected to be *Burkholderia* spp. due to their amplification using *B. vietnamiensis* and *B. ubonensis*-specific assays (Price et al., unpublished data).

### DNA Sequencing

When species determination discrepancies among the 122018, 266152, BurkDiff and TTS1 assays were observed, samples were subjected to multilocus sequence typing (MLST) [40] or 16 S sequencing [42]. Sequencing of the 266152 locus in *B. pseudomallei* Bp5706 was carried out by amplifying a ∼400 bp fragment that encompassed the 68 bp PCR product generated by the 266152 TaqMan assay to examine primer- or probe-binding mutations. Big Dye v3.1 chemistry (Applied Biosystems) was used for cycle sequencing. Sequencing products were denatured in Hi-Di formamide (Applied Biosystems) prior to electrophoresis on a 3130 or 3730 *xl* DNA Analyzer (Applied Biosystems).

## Results and Discussion

### Accuracy of 122018 and 266152 assays

**Figure 1:**
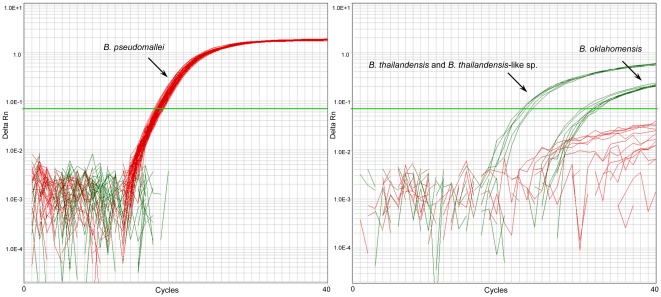
122018 TaqMan dual probe assay. Red, the *B. pseudomallei-*specific TaqMan probe amplifies only *B. pseudomallei* template; the non-*B.pseudomallei* TaqMan probe (green) amplifies well with *B. thailandensis* and *B. thailandensis-*like species and weakly with *B. oklahomensis* templates but not *B. pseudomallei*. Other *Burkholderia* spp. do not amplify with either probe.

**Figure 2:**
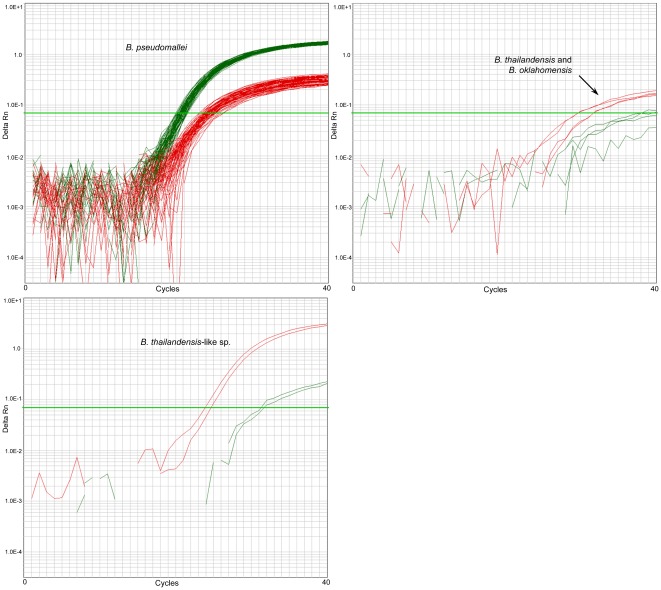
266152 TaqMan dual probe assay. Green, the *B. pseudomallei*-specific TaqMan probe preferentially amplifies *B. pseudomallei* template; the non-*B.pseudomallei* TaqMan probe (red) amplifies well in *B. thailandensis-*like species and weakly in *B. thailandensis* and *B. oklahomensis*. Other *Burkholderia* spp. do not amplify.

Accuracy is the measure of exactness of an analytical method, or the closeness of agreement between the measured value and the value that is accepted as a conventional true value or an accepted reference value [34], and is thus distinct from specificity and precision (see Methods S1 for definitions). We subjected the 122018 and 266152 SNP signatures to both *in silico* and laboratory screening to determine their accuracy towards *B. pseudomallei*. BLAST analysis was carried out at the 122018 and 266152 loci against all available *B. pseudomallei*, *B. mallei*, *B. thailandensis*, *B. thailandensis-*like, *B. vietnamiensis*, *B. oklahomensis*, *B. ubonensis* and *B. cepacia* genomes, which confirmed that only *B. pseudomallei* strains possessed the *B. pseudomallei-*specific allele at these loci. We then compared *in silico* and wet-bench genotypes using a panel of 13 whole genome-sequenced *Burkholderia* strains (Table S1). As predicted from *in silico* analysis, both assays amplified the *B. pseudomallei*-specific allele in all *B. pseudomallei* DNA samples, with no detectable amplification in the four *B. mallei* samples (results not shown). For 122018, both probes were specific to the appropriate species, with no cross-hybridization of the alternate probes (Figure 1). For 266152, *B. oklahomensis*, *B. thailandensis* and *B. thailandensis-*like species possessed some cross-hybridization with the *B. pseudomallei* probe but were distinguishable from *B. pseudomallei* due to preferential amplification of the non-*B. pseudomallei* probe (Figure 2).

### Specificity of 122018, 266152, BurkDiff and TTS1 assays

Following confirmation of assay accuracy, we screened DNA panels comprising 2,205 *Burkholderia* spp. (Table 2) and 127 non-*Burkholderia* species (Figure S1; Table S2) with the 122018, 266152, BurkDiff and TTS1 assays to determine their specificity. BurkDiff and TTS1 have previously demonstrated specificity for *B. pseudomallei* across moderately large DNA panels [13], [27]. As expected, the TTS1 assay showed excellent specificity for *B. pseudomallei*, although we detected one false-negative *B. pseudomallei* isolate, Bp1186 (original ID: SBCT-RF80-BP1, isolated from soil in Northeast Thailand). Further investigation of Bp1186 showed that it possessed a smaller genome than other *B. pseudomallei* strains, and lacked certain virulence loci, including *bimA* and other TTS1 loci besides *orf2*. These findings suggest that Bp1186 is probably unable to establish human infection (A. Tuanyok, unpublished data). Assay 266152 gave a single ambiguous call in *B. pseudomallei* Bp5706 (original ID: MSHR 1559) (0.05% of total samples) in which both probes amplified at the same time, albeit poorly. DNA sequence analysis of this isolate uncovered a second SNP (T/C) 6 bp upstream of the targeted SNP (result not shown). This SNP was within the probe binding site and thus altered probe-binding efficiency. No false-positives were detected using the TTS1 or 266152 assays.

BurkDiff was the only assay we examined that yielded no false-positives or false-negatives across our DNA panels. In contrast, using 122018, we observed six false-positives (0.3%) and no false-negatives. None of the other *B. pseudomallei-*specific assays gave detectable amplification of these six samples, indicating incorrect species assignment by 122018. Sequencing for 16 S rDNA and MLST confirmed one isolate as *B. vietnamiensis* and five as *B. ubonensis*. Twenty-seven other *B. ubonensis* and one other *B. vietnamiensis* did not amplify with the 122018 assay, suggesting variable prevalence of a *B. pseudomallei-*like locus within these species. All non-*Burkholderia* isolates were PCR-negative using the four assays.

Although BurkDiff provided the best speciation performance across our DNA panel, it was the most difficult assay to interpret due to heavy cross-hybridization between probes. Using pure *B. pseudomallei* templates, we observed a difference of C_T_ (ΔC_T_) of approximately 1, even with optimization measures employed for improving amplification efficiency (results not shown). Despite this very low ΔC_T_ we did not encounter an inconsistent genotyping call, indicating that this assay is robust in the presence of pure templates. Unlike BurkDiff, TTS1-positive genotypes were readily identifiable due to the single-probe format and amplification efficiency of this assay. However, one drawback of this single-probe format is that low-level cross contamination of *B. pseudomallei* DNA in non-*B. pseudomallei* templates can cause false-positive PCR results, and thus positive results must be interpreted with caution, especially when high C_T_ values are obtained. The 122018 and 266152 assays in their dual-probe format were not influenced by low-level *B. pseudomallei* contamination in *B. thailandensis*, *B. oklahomensis* and *B. thailandensis-*like templates, although low-level contamination of *B. pseudomallei* DNA in e.g. *B. ubonensis* samples remains problematic. Coupled with large ΔC_T_ values, the competitive dual-probe format enabled the most facile differentiation between *B. pseudomallei* and non-*B. pseudomallei* templates (Figures 1 and 2; Figures S2 and S3, Panel G).

Our results indicate that, with the exception of BurkDiff, no single genotyping method was 100% effective at speciating *B. pseudomallei*. Many promising molecular markers in *Burkholderia* spp. are homoplastic [18], [27]. Homoplastic markers may not be apparent when screening assays across relatively small (<1,000) isolate collections but can lead to false-positive and false-negative (as shown by our 122018 assay and the TTS1 assay) genotyping calls when assays are screened across larger and more diverse DNA panels. Additional mutations in probe- or primer-binding sites, such as observed with 266152, can cause aberrant genotyping results in otherwise promising species-specific signatures. In our study, we used a large amount of WGS data to minimize the probability of including homoplastic markers or SNPs with polymorphic flanking regions. As more WGS data for Burkholderiaceae are generated, this approach will continue to provide the most accurate speciation targets. Based on our findings, it is our recommendation that speciation of *B. pseudomallei* be based on at least two independent molecular markers, or a single molecular marker when latex agglutination testing is used, to ensure that false-negative and false-positive genotyping calls do not lead to erroneous species designations.

### Selectivity

The potential for near-neighbor contamination of DNA is a concern in complex specimens, such as those of environmental, clinical or forensic origin. We therefore performed a selectivity experiment (Methods S1) on the 122018 and 266152 assays to quantitatively assess their ability to detect minor *B. pseudomallei* components in the presence of near-neighbor DNA. We mixed *B. pseudomallei* and *B. thailandensis-*like templates in known ratios of 0∶100, 10∶90, 25∶75, 50∶50, 75∶25, 90∶10 and 100∶0, respectively. Both assays amplified the *B. pseudomallei*-specific probe (i.e. pure *B. pseudomallei* template) more efficiently than the alternate probe (i.e. pure *B. thailandensis-*like template) (Table S3; Figures 1 and 2). As expected, both alleles amplified when in the presence of mixed template (Table S3; Figures S2 and S3).

For the 122018 assay, all mixtures of both *B. pseudomallei* and *B. thailandensis-*like templates could be reliably distinguished from pure template at the lowest tested limit of selectivity (10%), and the 266152 assay was able to discriminate between pure *B. pseudomallei* and *B. thailandensis-*like template present at 10%. However, the *B. thailandensis-*like: *B. pseudomallei* mixtures at 50∶50, 25∶75 and 10∶90 ratios were indistinguishable from pure *B. pseudomallei* template using the 266152 assay (Figures S2 and S3; Table S3), indicating that the 266152 assay is insensitive to detecting *B. thailandensis-*like template when in the presence of 50% or greater *B. pseudomallei* template. Given the primary focus on *B. pseudomallei* in our study, we do not consider this result a failure in selectivity as we demonstrated that both assays yielded a significant difference (ΔC_T_ σ>2) between pure *B. pseudomallei* template and *B. pseudomallei* containing *B. thailandensis-*like template at a minor component of ≤10%.

Our experiments outline a rudimentary protocol for determining selectivity using TaqMan assays. Although beyond the scope of the current study, future studies should ideally examine lower minor component mixtures below 10% to determine the limit of selectivity for the 122018 and 266152 assays. Selectivity experiments using spiked environmental or clinical specimens, such as soil or sputum samples, would shed further light on the true selectivity performance of the 266152 and 122018 assays in the presence of PCR inhibitors or complex DNA constituents. Use of a single-probe approach may provide better detection of minor components than dual-probe format, although we do not recommend using the 266152 assay in a non-competitive format due to cross-hybridization of the probes, which may result in false-positive results.

### Limits of quantitation and detection (LoQ and LoD)

We calculated the lower LoQ and LoD (Tables S4 and S5) using pure DNA template for *B. pseudomallei* and *B. thailandensis-*like species. The LoQ was defined as the lowest level of DNA detected that provided an acceptable level of precision (i.e. 8/8 replicates amplified with a C_T_ standard deviation (σ) <0.8 from the mean C_T_), whereas LoD was measured as the concentration of analyte that gave rise to a signal significantly different from the negative control (i.e. at least 2/8 replicates amplified, irrespective of σ) [34]. We were not able to establish the upper LoD or LoQ as these values were not reached using our highest DNA amount of 40 ng/PCR. For the 122018 assay, the lower LoQ was ≥4×10^−5^ ng (≥40 fg, or 5 genomic equivalents (GEs)) and ≥400 fg (50 GEs) for *B. pseudomallei* and *B. thailandensis-*like templates, respectively, whereas the 266152 assay yielded LoQ values at ≥4 fg (∼0.5 GEs) and ≥4 ng (5×10^5^ GEs), respectively. The poor LoQ value for *B. thailandensis-*like template using the 266152 assay was surprising given that all eight replicates amplified at ≥400 fg (50 GEs). Using our LoQ criteria, this assay is unreliable for quantitating *B. thailandensis-*like DNA. The LoD of the 122018 assay was ≥4 fg (∼0.5 GEs) and ≥40 fg (5 GEs) for *B. pseudomallei* and *B. thailandensis-*like templates, respectively, and ≥4 fg total DNA (∼0.5 GEs) for both templates using the 266152 assay (Table S5). These results contrast with LoD values previously reported for the 266152 assay [35], which demonstrated a LoD of 10 GEs (see ‘Linearity’ below for further discussion on the 266152 results).

Although it is difficult to compare LoD values between studies due to experimental design differences (e.g. number of replicates tested, or differences in mastermix constituents, DNA quantitation, instruments, or thermal conditions), TaqMan probes theoretically have the capacity to reach 0.5 GEs (the equivalent of a single PCR target) in a well-designed assay. The TTS1 assay reportedly provides a LoD of 76 fg, or 10 GEs, in PCR and 122 fg (16 GEs) in spiked human blood [13], and the BurkDiff assay provides a LoD of 10 GEs in PCR [27]. Therefore, the LoD of our dual-probe assays are similar in performance to TTS1 and BurkDiff, particularly in the presence of *B. pseudomallei* template. As expected, the LoD and LoQ of the *B. thailandensis-*like template were less sensitive. Although not tested in the current study, the LoD and LoQ of near-neighbor templates, such as *B. thailandensis-*like species MSMB 43, could potentially be increased by using the non-*B. pseudomallei* probe by itself to avoid competition issues with the *B. pseudomallei-*specific probe.

### Linearity

We tested linearity of the 122018 and 266152 assays under controlled conditions (see Methods S1 for details) to establish the range of DNA amounts that enable accurate quantification [34]. Such information is valuable for quantifying the concentration of uncharacterized samples, or for determining the lowest concentration at which reliable genotyping data can be attained, particularly when DNA is limiting.

We did not reach the upper limit of linearity using the highest concentration of 40 ng DNA for either *B. pseudomallei* or *B. thailandensis-*like templates, indicating that the range of linearity for our assays is close to or greater than this amount. *B. thailandensis-*like template linearity lacked precision across DNA concentrations, particularly for assay 266152 (Table S6), indicating that the 266152 assay should not be used to quantify *B. thailandensis-*like DNA. The lower-limit of linearity for *B. pseudomallei*, based on 100% amplification across eight replicates and σ <0.8 between replicates, was 40 fg (5 GEs) and 4 fg (∼0.5 GEs) for the 122018 and 266152 assays, respectively. In other words, our data indicate that *B. pseudomallei* template can be accurately quantified down to 0.5 GEs (as also observed in ‘LoQ’ above), which is equivalent to a single PCR target, the theoretical limit of PCR detection. We have also determined the LoD of the 266152 assay as 10 GEs [35]; however, this value was based on 95% amplification success across 64 replicates, whereas the current study used only 25% amplification success across eight replicates and was thus less stringent. DNA quantitation varied between these two studies (NanoDrop spectrophotometric quantitation vs. normalization against a 16 S rDNA target), and it is possible that minor variations in DNA quantitation or dilution preparation influenced our quantitation results. Nevertheless, we have demonstrated that these dual-probe assays possess a large linearity range in the presence of pure *B. pseudomallei* templates and can thus be used to precisely quantify unknown samples across a wide range of DNA concentrations (Figure S4 and Table S6).

Determining linearity also allowed us to calculate the PCR efficiency of the 122018 and 266152 assays. For the 122018 assay, PCR efficiency was 91% and 89% for *B. pseudomallei* and *B. thailandensis-*like templates, respectively. The efficiency of the 266152 assay was higher, at 97% and 94% for *B. pseudomallei* and *B. thailandensis-*like templates, respectively (Table S6). In contrast, the efficiency of the TTS1 assay has been reported at 99% for *B. pseudomallei* [13]. However, the linear range used in this study was much more restrictive than ours due to the smaller number of DNA concentrations included in the linear dynamic range, so comparison of PCR efficiencies between studies must be prudently interpreted. It remains to be determined whether PCR efficiency for the 122018 and 266152 assays could be improved by using single-probe format, as is used in the TTS1 assay.

### Robustness and Ruggedness

Robustness and ruggedness are oft-neglected aspects of assay performance, despite their inter-laboratory importance. We therefore assessed these criteria for the 122018 and 266152 assays using multiple AB7900HT instruments (ruggedness), and TaqMan probe and Universal Master Mix (Applied Biosystems) reagent lots, types of commercial mastermixes (Universal and Genotyping Master Mixes; Applied Biosystems) and annealing/extension temperatures (robustness) to determine those features most critical to inter-laboratory assay transfer.

Comparison of commercial mastermixes with the 266152 and 122018 assays yielded unexpected results. The TaqMan Genotyping Master Mix (Applied Biosystems) showed poor amplification with the 122018 and 266152 assays (results not shown), despite its purported suitability for SNP genotyping applications (http://products.invitrogen.com/ivgn/product/4371355). Due to the proprietary nature of most commercial mastermixes, we were unable to establish the component difference between the Genotyping and Universal Master Mixes. However, the BurkDiff assay does not amplify using the TaqMan Universal Master Mix (results not shown), suggesting that neither mix is ideal for all SNP genotyping applications. In contrast, we did not observe differences in precision or accuracy of amplification using three different AB7900HT instruments, nor did we identify performance differences among two probe and four TaqMan Universal Master Mix reagent lots (results not shown), suggesting that our TaqMan assays can be reliably reproduced on this platform using our reaction conditions. We did not test the assays on different real-time PCR platforms. Thus, further studies are required to determine their suitability on different real-time PCR instruments and across other commercial mastermixes.

Although our TaqMan probes were specifically designed with an optimal annealing/extension temperature of 60°C, inter-laboratory differences in thermal block temperatures can potentially influence genotyping calls. We therefore tested the robustness of the 122018 and 266152 assays across multiple annealing/extension temperatures and observed their effect on accurately and precisely calling correct genotypes. Overall, annealing/extension temperatures at 57.5 and 62.5°C still amplified templates and gave correct genotyping calls, although we did observe differences in robustness at these altered temperatures, particularly when the *B. thailandensis-*like template was assessed (Table S7). For the 122018 assay, the 57.5°C annealing/extension temperature was comparable in performance to 60°C, whereas 62.5°C exhibited more amplification failures than at the lower annealing/extension temperatures. In contrast, the 62.5°C and 60°C annealing/extension temperatures for assay 266152 were comparable in performance, whereas 57.5°C replicates gave poorer replicate success, especially in the presence of *B. thailandensis-*like template. The ΔC_T_ values at 57.5°C and 62.5°C were smaller than those at 60°C, demonstrating that 60°C is indeed the optimal temperature (Table S7) although they did not result in an increase in cross-hybridization. Importantly, no erroneous genotypes were observed at either the reduced or elevated temperature for either assay, indicating that these assays are tolerant to thermal block variations, but deviations from 60°C will result in a reduction in assay robustness and thus assays should ideally be optimized on each individual instrument.

### Conclusions

*B. pseudomallei* is an important pathogen from a clinical, environmental and forensic stance. Correct identification of *B. pseudomallei* requires molecular characterization due to shortcomings with phenotypic speciation techniques. Identification and quantification of *B. pseudomallei* from pure cultures through to complex soil or sputum samples is dependent on a thorough understanding of the limits of species-specific assays. Despite the plethora of *B. pseudomallei* assays available, few if any have been subjected to rigorous performance criteria. We therefore identified, designed and thoroughly tested two novel *B. pseudomallei-*specific molecular assays, 122018 and 266152, by subjecting them to several parameters: accuracy, specificity, precision, selectivity, LoQ, LoD, linearity, ruggedness and robustness. The accuracy and specificity of the 122018 and 266152 assays were compared with those of two well-established *B. pseudomallei* assays, BurkDiff and TTS1. BurkDiff provided the best specificity, with no false-positives or false-negatives detected across 2,205 *Burkholderia* samples and 127 common soil, water or clinical species. Assay 266152 provide a single ambiguous genotyping call for a *B. pseudomallei* isolate, the TTS1 assay gave one false-negative result, and the 122018 assay gave six false-positive calls in certain *B. ubonensis* and *B. vietnamiensis* strains.

As these four assays become more widely used, we have no doubt that false-positives and false-negatives will be encountered; however, our work suggests that this will be a rare occurrence and false conclusions will be further minimized by including more than one of these assays for speciation. Additional specificity testing of TTS1, BurkDiff, 122018 and 266152 assays on different *Burkholderia* species is important as such strains will provide more informative false-positive rates due to their genetic relatedness to *B. pseudomallei*. Likewise, further testing of *B. pseudomallei* isolates using these four assays will increase the accuracy of false-negative rates. Our assays demonstrated comparable LoD and LoQ performance to the current ‘gold standard’ *B. pseudomallei* typing techniques. Although assay parameters like ruggedness, robustness and selectivity are not typically examined when developing and validating molecular assays, we anticipate that our methods will provide a framework for future studies where quantitative measures of comparative assay performance are paramount. Last, accurate standardization of input DNA is a crucial component of assay performance yet is difficult for complex environmental or clinical specimens where *B. pseudomallei* is usually isolated, due to the non-homogeneous nature of these samples, the presence of PCR inhibitors or the abundance of non-*Burkholderia* DNA. While the tests of assay performance included here measure the effects of many potential variables, this list is not comprehensive. As such, users should be aware that other untested factors that might be encountered when samples are extracted from complex environments may have an impact on assay performance.

## Supporting Information

Methods S1 Supplemental materials and methods.
(DOC)

Figure S1 266152 assay specificity results against non-*Burkholderia* yeast, fungal and bacterial species and the *B. pseudomallei* sample, 104 (see Table S2 for the list of organisms). Only the *B. pseudomallei* sample, 104, amplified with this assay (red and green amplification curves). All samples were run in duplicate. TTS1 and 122018 assays performed identically to 266152, with no amplification in non-*Burkholderia* species (results not shown).
(DOC)

Figure S2 Selectivity performance of the 122018 assay using varying mixtures of *Burkholderia pseudomallei* 104 (Bp; green) and *B. thailandensis-*like MSMB 43 (Bh; red) DNA. A, 0∶100 Bp∶Bh; B, 10∶90 Bp∶Bh; C, 25∶75 Bp∶Bh, D, 50∶50 Bp∶Bh, E, 75∶25 Bp∶Bh; F, 90∶10 Bp∶Bh, G, 100∶0 Bp∶Bh. All mixture ratios could be differentiated from pure Bp or Bh template (see Table S3 for details).
(DOC)

Figure S3 Selectivity performance of the 266152 assay using varying mixtures of *Burkholderia pseudomallei* 104 (Bp; green) and *B. thailandensis*-like MSMB 43 (Bh; red) DNA. A, 0∶100 Bp∶Bh; B, 10∶90 Bp∶Bh; C, 25∶75 Bp∶Bh, D, 50∶50 Bp∶Bh, E, 75∶25 Bp∶Bh; F, 90∶10 Bp∶Bh, G, 100∶0 Bp∶Bh. Note that in D, E and F, the standard deviation (σ) between curves falls below our accepted threshold (see Table S3 for details), indicating that DNA mixtures containing up to 50% Bh DNA cannot reliably be differentiated from pure Bp template (G) with this assay.
(DOC)

Figure S4 Range of linearity for *B. pseudomallei* 122018 and 266152 TaqMan real-time PCR assays.
(DOC)

Table S1 Determination of 122018 and 266152 assay accuracy by comparison of *in silico* and TaqMan real-time PCR “wet-bench” single-nucleotide polymorphism results.
(DOC)

Table S2 Non-*Burkholderia* species of clinical, environmental or forensic importance tested against the TTS1, 122018 and 266152 TaqMan assays for specificity (also see Figure S1). BurkDiff was not tested with this panel as it has been previously screened against a similar panel of 390 non-*Burkholderia* species [27]. Bacteria, no shading; fungi, light grey; yeasts, dark grey.
(DOC)

Table S3 Selectivity results for *B. pseudomallei* 122018 and 266152 assays.
(DOC)

Table S4 Limit of Quantitation (LoQ) for the 122018 and 266152 assays.
(DOC)

Table S5 Limit of Detection (LoD) for the 122018 and 266152 assays.
(DOC)

Table S6 Range of linearity for *B. pseudomallei* 122018 and 266152 TaqMan assays.
(DOC)

Table S7 Robustness summary for 122018 and 266152 TaqMan assays.
(DOC)
