# Supplementary material for: Development and Validation of Burkholderia pseudomallei-Specific Real-Time PCR Assays for Clinical, Environmental or Forensic Detection Applications
Source: PLoS One. 2012 May 18;7(5):e37723. doi: 10.1371/journal.pone.0037723 (PMC3356290; doi:10.1371/journal.pone.0037723)
Supplement: Methods S1 — Supplemental materials and methods. (DOC) [file pone.0037723.s001.doc]

The 122018 and 266152 assays were subjected to multiple assay performance criteria to determine their suitability for use under a range of potential conditions. The following parameters were tested: accuracy, specificity, precision, selectivity, limit of quantitation (LoQ), limit of detection (LoD), linearity, ruggedness and robustness. Our definition of these parameters was modified for genotyping analysis based on the standardized definitions described elsewhere [1]. Unless otherwise specified, *B. pseudomallei* 104 and *B. thailandensis*-like MSMB43 were used as DNA controls for *B. pseudomallei* and non*-B. pseudomallei* templates, respectively.

**Accuracy**

Accuracy is the measure of exactness of an analytical method, or the closeness of agreement between the measured value and the value that is accepted as a conventional true value or an accepted reference value [1]. For our investigation, we determined the accuracy of the 122018 and 266152 *B. pseudomallei* SNPs by comparing their *in silico* SNP state to “wet-bench” genotyping results (Table S1). All assays behaved as expected according to *in silico* predictions. See “Results and Discussion – Accuracy of 122018 and 266152 assays” in the main manuscript for details.

**Specificity**

We defined specificity as the ability to measure the analyte of interest to the exclusion of other relevant components. We performed an assessment of assay specificity by screening a comprehensive collection of *Burkholderia* DNA samples against TTS1 [2], BurkDiff [3], 122018, and 266152 assays to determine their ability to accurately speciate *B. pseudomallei*. All assays were screened across the 2,205 *Burkholderia* samples, which encompasses at least ten different *Burkholderia* species (Table 2). DNA was quantified on the NanoDrop 8000 spectrophotometer and normalized to 1ng/uL. 16S rDNA detection [4] was implemented on *Burkholderia* samples that did not amplify using any of the *B. pseudomallei-*specific assays (122018, 266152, TTS1 and BurkDiff) to confirm DNA quality.

We also screened the 122018 and 266152 assays across other bacterial and fungal species that are potentially found in the same niches as *B. pseudomallei* to further validate specificity of these assays (Table S2). 16S rDNA detection was also used to confirm DNA quality in all organisms, including in fungal and yeast species.

**Precision**

Precision is the degree of agreement between quantity values obtained by repeated replicate measurements of a homogeneous sample [1]. Precision can be affected by several variables including template quality and amount, PCR conditions (e.g. annealing temperature), reagent lot variability, instrumentation, and technical/mechanical errors. For our study, we tested precision in a controlled experiment, wherein most of these variables were minimized except for the amount of DNA, for which a range was tested. We measured precision in two ways: 1) using serial dilution experiments, and; 2) testing multiple templates over multiple PCR plates.

*Serial dilution experiments*

Precision of PCR replicates was extensively measured in the LoD, LoQ and linearity parameters outlined below by examining the standard deviation (σ) of ten-fold serially diluted DNA templates across as many as 16 replicates. An outlier was defined as a single replicate amplifying with a cycles to threshold (C_T_) σ>0.8 from the mean C_T_ for those replicates. To account for the effects of pipetting error on precision, C_T_ averages and σ were examined for a) all amplifying replicates, and; b) all amplifying replicates minus any single outliers. A loss of precision for a dilution point was defined as replicates with C_T_ σ>0.8 or with amplification failures in any of the replicates. The precision data for these experiments are shown in Tables S4, S5 and S6.

*Multiple templates over multiple plates*

The 122018 and 266152 assays were replicated three independent times to determine their precision (i.e. their degree of agreement) between these replicate measurements. We measured precision for both probes within each assay by temporal testing of the *B. pseudomallei* 122018 and 266152 assays against a panel of 67 *B. pseudomallei*, *B. mallei*, *B. thailandensis* and *B. oklahomensis* isolates. Repeat testing against the 67 isolates (results not shown) gave no inconsistencies between the probes and their likely genotypes.

**Selectivity**

Selectivity is defined as the extent in which an analyte can be identified in a mixture without interference from other components [1]. In PCR, selectivity can be affected by the ratio of target to background, near neighbor contaminants in the background, the ratio of multiple target templates, the quality of individual target templates, amount of target template, cross hybridization between probes, and differences in affinities between the two probes in a single assay. In particular, the potential for near neighbor contaminants of *B. pseudomallei* in environmental and forensic sampling is a real concern for selectivity.

We determined 122018 and 266152 selectivity by comparing pure template with a range of mixed DNA amounts of *B. pseudomallei* and *B. thailandensis-*like MSMB43 (Figures S2 and S3). Ratios tested were 0:100, 10:90, 25:75, 50:50, 75:25, 90:10 and 100:0. For all ratios, a total DNA amount of 2ng was used in each PCR. Four replicates for each sample and two negative controls were run for each assay. The average ∆C_T_ difference between the *B. pseudomallei*-specific and non *B. pseudomallei*-specific probes was calculated for all mixtures (Table S3). The ∆C_T_’s for the mixed samples were compared with the ∆C_T_’s for pure *B. pseudomallei* and *B. thailandensis-*like control samples to see if they were measurably different. Samples with a σ>2 compared with the pure template DNA control DNA were considered measurably different and thus indicated ‘mixed’ sample status.

**LoQ and LoD**

LoQ was determined by identifying the lowest and highest amount of *B. pseudomallei* or *B. thailandensis-*like MSMB43 DNA that could be measured with an acceptable level of specificity and precision [1]. Our LoQ experiments involved testing the *B. pseudomallei*-specific assays against eight replicates across 16 serially diluted DNA amounts, ranging from 40ng to 4x10^-14^ng. We used a σ<0.8 parameter on all replicate C_T_s to determine LoQ (see ‘Precision’ above); any replicates falling outside this σ were considered imprecise and thus exceeded the LoQ.

Using the LoQ data as a guide, the LoD was determined as the amount of analyte that gave rise to a signal significantly different from the negative control [1]. From the LoQ data, we chose multiple dilution points ranging from 100% successful LoQ amplification (i.e. all eight LoQ replicates amplified), potentially spotty amplification (<8 replicates amplified), and 100% failed amplification (see ‘Precision’ above). Eight replicates of each dilution point for *B. pseudomallei* and *B. thailandensis-*like MSMB43 were run for LoD. We defined the LoD as the lowest range of DNA amounts that gave 25% or better amplification across eight replicates. See Tables S4 and S5 for LoQ and LoD results, respectively.

We calculated genomic equivalents (GEs) assuming a 7.2Mbp genome size for *B. pseudomallei* and *B. thailandensis*-like MSMB43 and an average molecular weight per bp of 660 g/mol.

**Linearity**

We defined linearity as the ability to elicit results that are directly, or by a well defined mathematical transformation, proportional to the amount of DNA in the sample. The range of linearity of a PCR assay is a function of amplification efficiency, which is measured by the rate at which a PCR amplicon is generated. 100% efficiency is defined as the doubling of PCR amplicon during the geometric phase of its PCR amplification, resulting in a 10-fold increase of the PCR amplicon every 3.32 cycles [5]. Thus, maximum quantitative accuracy occurs in assays that function at or near 100% PCR efficiency.

Factors that can affect real-time PCR efficiency and therefore linearity include: a) extreme high and low starting target amounts that may result in unexpected and/or non-reproducible C_T_ values and reduced precision among replicates; b) inefficient primer/probe hybridization attributable to poor assay design, additional SNPs in primer or probe sites, competition from the mismatched probe, or suboptimal annealing temperatures; and c) non-assay related effects, such as target template quality, PCR conditions, commercial Master Mix variability, instrumentation, technical/mechanical errors, or poor primer/probe batch synthesis. Where possible, we minimized the effects of these factors through assay optimization and strict monitoring of reagent lots.

For this study, we examined assay linearity over a range of DNA amounts using the LoQ data, as described above. We used the following cutoffs to define data points within the linear range: slope (between -3.3 and -3.7), replicate number (≥25% amplification success) and R^2^ values (> 0.997). We also determined the linear range using the above parameters in addition to an σ cutoff of ≤0.8 (see ‘Precision’ above); this more stringent linear range should be used when <8 replicates are used. For all linear plots, PCR efficiency (E_X_) was calculated using the formula: E_X_ = 10^(-1/slope)^ -1 [6] (<http://www3.appliedbiosystems.com/cms/groups/mcb_marketing/documents/generaldocuments/cms_040377.pdf>) (Figure S4).

Ranges of linearity for the *B. pseudomallei* canSNP assays can be found in Table S6. Applied Biosystems guidelines (see URL above) suggest using at least five dilution points to generate a slope calculation for a given assay. All assays met this criterion. However, when σ<0.8 was factored into linearity, this range decreased dramatically for the *B. thailandensis-*like MSMB43 template for all assays (Table S6; Figure S4). For 122018, factoring in the SDs of the eight replicates still provided five dilution points; however, the SD values dramatically decreased the range of linearity for 266152 non-*B.pseudomallei* probes by one dilution point. It should be noted that the purpose of the *B. pseudomallei* canSNP assays was not specifically to amplify non-*B. pseudomallei* targets; nevertheless, caution should be applied when interpreting linearity data from non-*B. pseudomallei* templates, and it is recommended that a large number of replicates (e.g. eight) for both the standard curve and for unknown samples would need to be included to provide the most accurate and reliable data.

**Ruggedness and Robustness**

Ruggedness is defined as the constancy of results when external factors, such as different instruments, laboratories, analysts or reagents, are deliberately varied [1]. In this study, we examined the ruggedness of our assays by a) testing their performance on two AB7900HT thermal cyclers, and; b) using multiple TaqMan probe and Universal Master Mix reagent lots. No differences in precision and accuracy for either *B. pseudomallei* assays were observed using different reagent lots or instruments, and all allele calls were 100% accurate (results not shown), indicating that our assays have a high degree of ruggedness.

Robustness, which differs from ruggedness in that internal rather than external features of the experiment are altered [1] was examined by altering the annealing/extension temperatures during thermocycling ±2.5^o^C from the default of 60^o^C. We tested this particular robustness parameter due to common differences in PCR equipment between laboratories, which may vary with respect to preciseness of block temperature. Each assay was tested across a ten-fold serial dilution series containing four points. These four points were selected for each genotype for each assay based upon the results of the LoD experiment: the lowest two points with successful amplification, and two 10-fold dilutions with spotty amplification. Four replicates of each dilution point for *B. pseudomallei* and *B. thailandensis-*like DNA were run. Eight NTCs for each assay were also run. Robustness was measured by three parameters: the average ΔC_T_ between the *B. pseudomallei* and non-*B. pseudomallei* probes, the C_T_ σ, and the amplification stability of the four replicates at each dilution point. A loss of precision for a dilution point was defined as replicates with C_T_ σ>0.8 or with amplification failures in any of the four replicates. Robustness results are summarized in Table S7.

**References**

1. Araujo P (2009) Key aspects of analytical method validation and linearity evaluation. J Chromatogr B Analyt Technol Biomed Life Sci 877: 2224-2234.

2. Novak RT, Glass MB, Gee JE, Gal D, Mayo MJ, et al. (2006) Development and evaluation of a real-time PCR assay targeting the type III secretion system of *Burkholderia pseudomallei*. J Clin Microbiol 44: 85-90.

3. Bowers JR, Engelthaler DM, Ginther JL, Pearson T, Peacock SJ, et al. (2010) BurkDiff: a real-time PCR allelic discrimination assay for *Burkholderia pseudomallei* and *B. mallei*. PLoS One 5: e15413.

4. Nadkarni MA, Martin FE, Jacques NA, Hunter N (2002) Determination of bacterial load by real-time PCR using a broad-range (universal) probe and primers set. Microbiology 148: 257-266.

5. Svanvik N, Stahlberg A, Sehlstedt U, Sjoback R, Kubista M (2000) Detection of PCR products in real time using light-up probes. Anal Biochem 287: 179-182.

6. Vaerman JL, Saussoy P, Ingargiola I (2004) Evaluation of real-time PCR data. J Biol Regul Homeost Agents 18: 212-214.
