# Supplementary figures and images for: Development and Validation of Burkholderia pseudomallei-Specific Real-Time PCR Assays for Clinical, Environmental or Forensic Detection Applications
Source: PLoS One. 2012 May 18;7(5):e37723. doi: 10.1371/journal.pone.0037723 (PMC3356290; doi:10.1371/journal.pone.0037723)

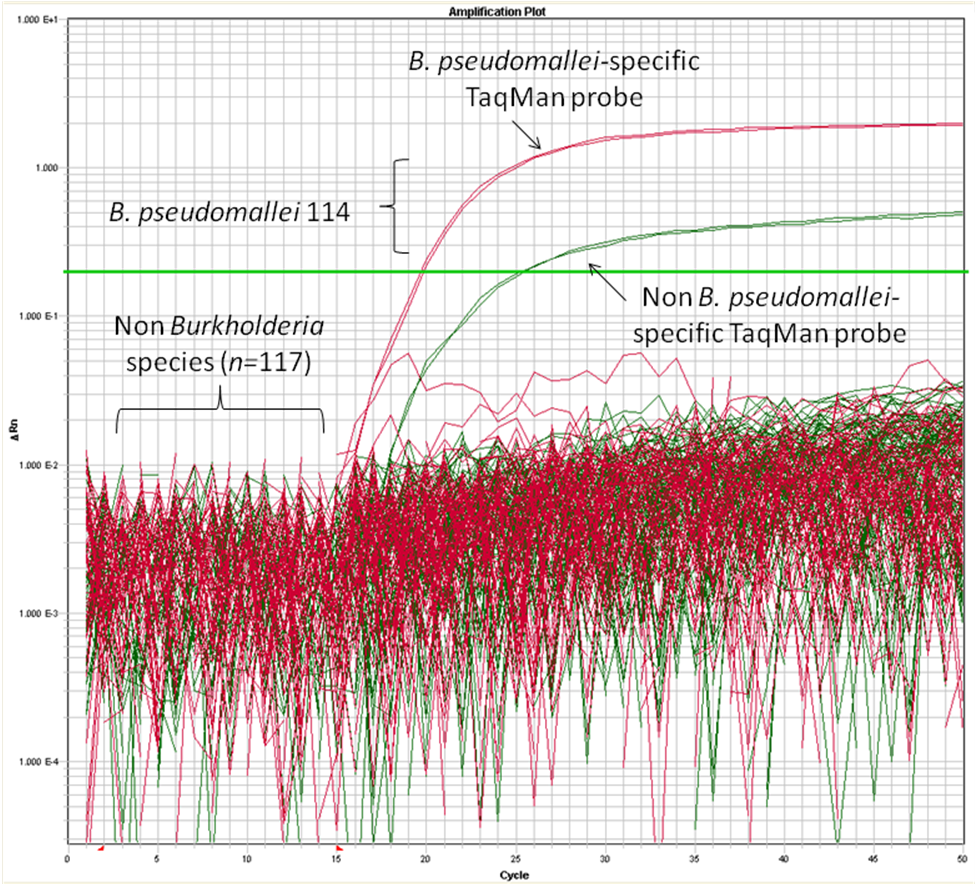

Supplement: Figure S1 — 266152 assay specificity results against non-Burkholderia yeast, fungal and bacterial species and the B. pseudomallei sample, 104 (see Table S2 for the list of organisms). Only the B. pseudomallei sample, 104, amplified with this assay (red and green amplification curves). All samples were run in duplicate. TTS1 and 122018 assays performed identically to 266152, with no amplification in non-Burkholderia species (results not shown). (DOC) [file pone.0037723.s002.doc]

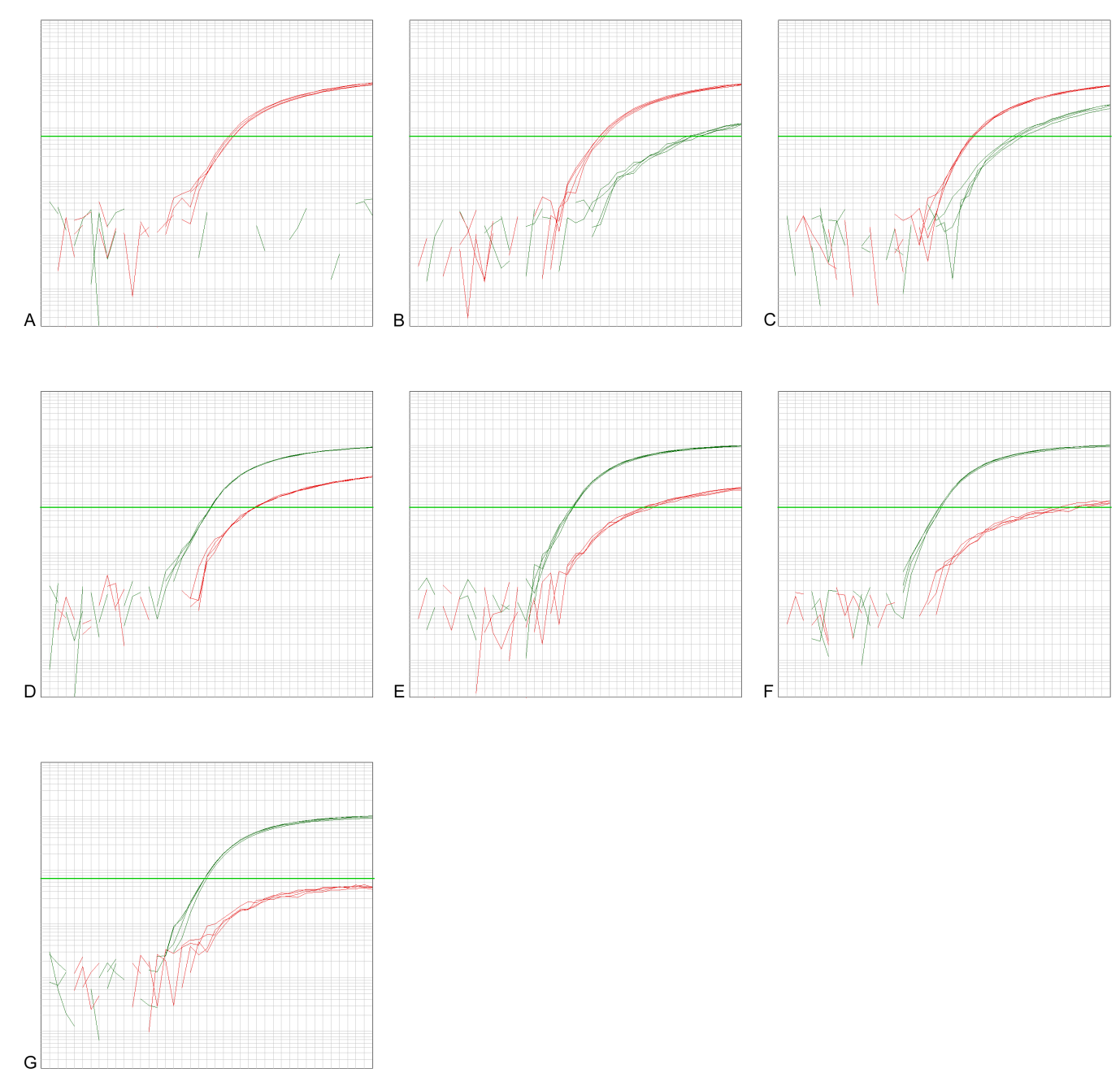

Supplement: Figure S2 — Selectivity performance of the 122018 assay using varying mixtures of Burkholderia pseudomallei 104 (Bp; green) and B. thailandensis-like MSMB 43 (Bh; red) DNA. A, 0∶100 Bp∶Bh; B, 10∶90 Bp∶Bh; C, 25∶75 Bp∶Bh, D, 50∶50 Bp∶Bh, E, 75∶25 Bp∶Bh; F, 90∶10 Bp∶Bh, G, 100∶0 Bp∶Bh. All mixture ratios could be differentiated from pure Bp or Bh template (see Table S3 for details). (DOC) [file pone.0037723.s003.doc]

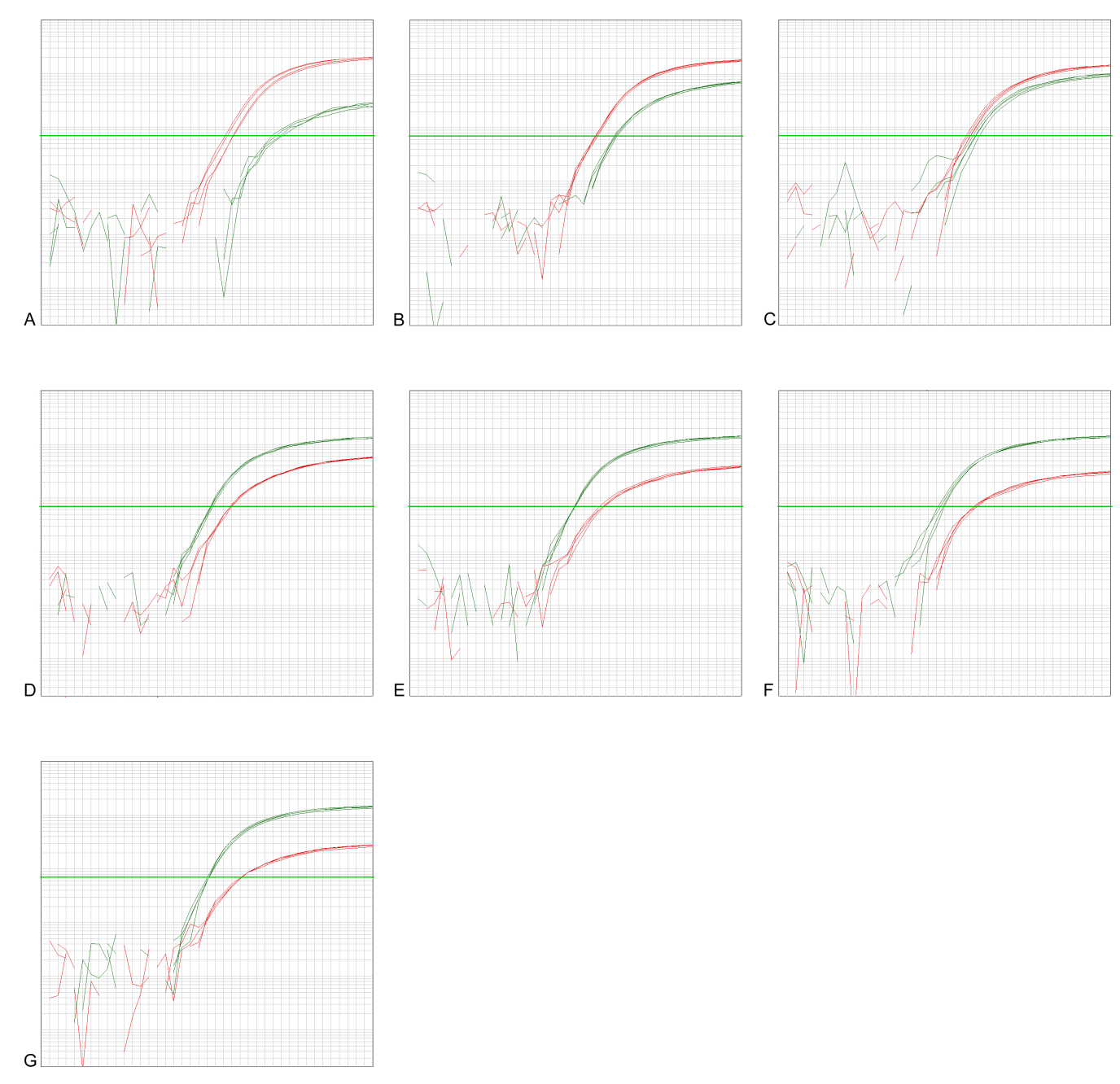

Supplement: Figure S3 — Selectivity performance of the 266152 assay using varying mixtures of Burkholderia pseudomallei 104 (Bp; green) and B. thailandensis-like MSMB 43 (Bh; red) DNA. A, 0∶100 Bp∶Bh; B, 10∶90 Bp∶Bh; C, 25∶75 Bp∶Bh, D, 50∶50 Bp∶Bh, E, 75∶25 Bp∶Bh; F, 90∶10 Bp∶Bh, G, 100∶0 Bp∶Bh. Note that in D, E and F, the standard deviation (σ) between curves falls below our accepted threshold (see Table S3 for details), indicating that DNA mixtures containing up to 50% Bh DNA cannot reliably be differentiated from pure Bp template (G) with this assay. (DOC) [file pone.0037723.s004.doc]

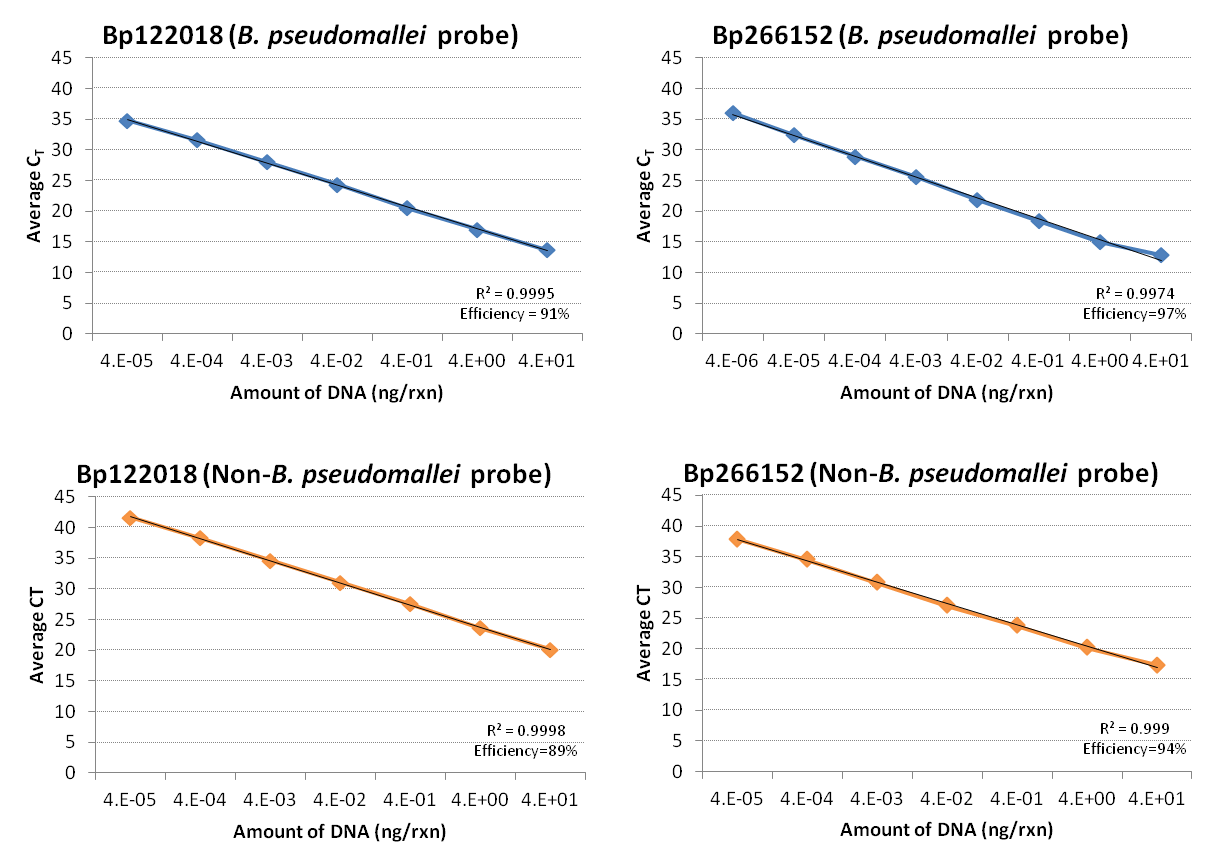

Supplement: Figure S4 — Range of linearity for B. pseudomallei 122018 and 266152 TaqMan real-time PCR assays. (DOC) [file pone.0037723.s005.doc]
