# Supplementary material for: Development and Validation of Burkholderia pseudomallei-Specific Real-Time PCR Assays for Clinical, Environmental or Forensic Detection Applications
Source: PLoS One. 2012 May 18;7(5):e37723. doi: 10.1371/journal.pone.0037723 (PMC3356290; doi:10.1371/journal.pone.0037723)
Supplement: Table S1 — Determination of 122018 and 266152 assay accuracy by comparison of in silico and TaqMan real-time PCR “wet-bench” single-nucleotide polymorphism results. (DOC) [file pone.0037723.s006.doc]

| **Species** | **Original ID** | **SNP122018** | | **SNP266152** | |
| --- | --- | --- | --- | --- | --- |
|  |  | ***In silico*^a^** | **Real-time PCR** | ***In silico*^a^** | **Real-time PCR** |
| *B. pseudomallei* | 305 | T | T | T | T |
| *B. pseudomallei* | 346 | T | T | T | T |
| *B. pseudomallei* | Pasteur 52237 | T | T | T | T |
| *B. pseudomallei* | 7894 | T | T | T | T |
| *B. pseudomallei* | B7210 | T | T | T | T |
| *B. pseudomallei* | 9 | T | T | T | T |
| *B. mallei* | NCTC 10245 | NA | NA | NA | NA |
| *B. mallei* | ATCC 23344 | NA | NA | NA | NA |
| *B. mallei* | NCTC 10229 | NA | NA | NA | NA |
| *B. mallei* | NCTC 10247 | NA | NA | NA | NA |
| *B. ubonensis* | NCTC 13147 | NA | NA | NA | NA |
| *B. vietnamiensis* | G4 | NA | NA | NA | NA |
| *B. oklahomensis* | C6786 | C | C | C | C |
| *B. oklahomensis* | E0147 | C | C | C | C |
| *B. thailandensis* | E264 | C | C | C | C |
| *B. thailandensis*-like | MSMB43 | C | C | C | C |

^a^ Whole genome sequence (WGS) data was used to determine *in silico* genotyping calls. WGS data are available at: <http://img.jgi.doe.gov/cgi-bin/w/main.cgi>.
