# Supplementary material for: Development and Validation of Burkholderia pseudomallei-Specific Real-Time PCR Assays for Clinical, Environmental or Forensic Detection Applications
Source: PLoS One. 2012 May 18;7(5):e37723. doi: 10.1371/journal.pone.0037723 (PMC3356290; doi:10.1371/journal.pone.0037723)
Supplement: Table S2 — Non-Burkholderia species of clinical, environmental or forensic importance tested against the TTS1, 122018 and 266152 TaqMan assays for specificity (also see Figure S1). BurkDiff was not tested with this panel as it has been previously screened against a similar panel of 390 non-Burkholderia species [27]. Bacteria, no shading; fungi, light grey; yeasts, dark grey. (DOC) [file pone.0037723.s007.doc]

| **Organism** | **No. Strains** | **Organism** | **No. strains** |
| --- | --- | --- | --- |
| *Achromobacter xylosoxidans* | 1 | *Ralstonia eutropha* | 1 |
| *Acinetobacter baumannii** | 3 | *Ralstonia solanacearum** | 2 |
| *Aeromonas hydrophila* | 1 | *Rhizobium radiobacter** | 2 |
| *Bacillus anthracis** | 1 | *Rothia mucilaginosa* | 1 |
| *Bacillus cereus** | 1 | *Saccharopolyspora rectivirgula* | 1 |
| *Bacteroides fragilis* | 1 | *Salmonella infantis* | 1 |
| *Chryseobacterium indologenes* | 1 | *Salmonella typhimurium** | 1 |
| *Corynebacterium diphtheriae** | 1 | *Serratia marcesens* | 1 |
| *Corynebacterium pseudotuberculosis* | 1 | *Staphlyococcus epidermidis** | 2 |
| *Corynebacterium* sp*.* | 1 | *Staphylococcus aureus** | 4 |
| *Cupriavidus* sp. * | 8 | *Staphylococcus haemolyticus** | 1 |
| *Eikenella corrodens* | 1 | *Staphylococcus hominis** | 1 |
| *Enterobacter aerogenes* | 1 | *Staphylococcus hyicus* | 1 |
| *Enterobacter cloacae** | 5 | *Staphylococcus intermedius* | 1 |
| *Enterococcus faecalis** | 2 | *Staphylococcus sciuri* | 1 |
| *Enterococcus* sp. | 2 | *Stenotrophomonas maltophilia** | 4 |
| *Escherichia coli** | 4 | *Streptococcus agalactiae** | 2 |
| *Francisella tularensis** | 1 | *Streptococcus alactolyticus* | 1 |
| *Fusobacterium necrophorum* | 1 | *Streptococcus anginosus* | 1 |
| *Fusobacterium nucleatum* | 1 | *Streptococcus mitis** | 1 |
| *Haemophilus influenzae** | 5 | *Streptococcus mutans** | 1 |
| *Haemophilus parainfluenzae* | 1 | *Streptococcus pneumoniae** | 3 |
| *Klebsiella oxytoca** | 1 | *Streptococcus pyogenes** | 3 |
| *Klebsiella pneumoniae** | 6 | *Streptococcus viridans* | 3 |
| *Legionella pneumophila** | 1 | *Yersinia pestis** | 1 |
| *Listeria monocytogenes** | 1 | *Yersinia pseudotuberculosis** | 1 |
| *Moraxella catarrhalis** | 6 | *Absidia corymbifera* | 1 |
| *Mycobacterium avium** | 2 | *Rhizopus oryzae* | 1 |
| *Mycoplasma pneumoniae* | 1 | *Histoplasma* sp.* | 1 |
| *Neisseria meningitidis* | 1 | *Coccidioides immitis** | 1 |
| *Parabacteroides distasonis* | 1 | *Candida albicans** | 1 |
| *Pasteurella multocida* | 1 | *Candida parapsilosis* | 1 |
| *Peptococcus niger* | 1 | *Aspergillus niger* | 1 |
| *Porphyromonas gingivalis* | 1 | *Saccharomyces cerevisiae* | 1 |
| *Pseudomonas aeruginosa** | 7 | *Cryptococcus neoformans* | 1 |
| *Pseudomonas fluorescens** | 2 | **Total** | **127** |

*These species have been previously tested for 266152 assay specificity using different strains [35].
