# Supplementary material for: Development and Validation of Burkholderia pseudomallei-Specific Real-Time PCR Assays for Clinical, Environmental or Forensic Detection Applications
Source: PLoS One. 2012 May 18;7(5):e37723. doi: 10.1371/journal.pone.0037723 (PMC3356290; doi:10.1371/journal.pone.0037723)
Supplement: Table S3 — Selectivity results for B. pseudomallei 122018 and 266152 assays. (DOC) [file pone.0037723.s008.doc]

| **Assay 122018** | | | | | |  |  |
| --- | --- | --- | --- | --- | --- | --- | --- |
| **Mixture ratio^a^** | **Bp specific probe^b^** | | **Non-Bpspecific probe^b^** | | **Bp C_T_ minus Non-Bp C_T_ (∆C_T_)** | **Bh σ ∆C_T_^c^** | **Bp σ ∆C_T_^d^** |
|  | Mean C_T_ | σ | Mean C_T_ | σ |  |  |  |
| Bh:Bp 100:0 | 40.00 | N/A | 22.85 | 0.28 | -17.15 | N/A | 26.46 |
| Bh:Bp 90:10 | 34.72 | 0.66 | 23.10 | 0.28 | -11.62 | 3.91 | 22.55 |
| Bh:Bp 75:25 | 29.01 | 0.65 | 23.34 | 0.05 | -5.67 | 8.12 | 18.35 |
| Bh:Bp 50:50 | 20.37 | 0.07 | 25.92 | 0.27 | 5.55 | 16.05 | 10.41 |
| Bh:Bp 25:75 | 19.57 | 0.14 | 28.63 | 0.67 | 9.06 | 18.54 | 7.93 |
| Bh:Bp 10:90 | 19.46 | 0.10 | 34.98 | 1.87 | 15.52 | 23.10 | 3.36 |
| Bh:Bp 0:100 | 19.73 | 0.13 | 40.00 | N/A | 20.27 | 26.46 | N/A |
| NTC | -- | -- | -- | -- | -- | -- | -- |
| **Assay 266152** | | | | | |  |  |
| Bh:Bp 100:0 | 28.25 | 0.84 | 22.65 | 0.56 | -5.61 | N/A | 6.75 |
| Bh:Bp 90:10 | 24.83 | 0.23 | 22.47 | 0.17 | -2.36 | 2.30 | 4.45 |
| Bh:Bp 75:25 | 23.73 | 0.32 | 22.88 | 0.24 | -0.85 | 3.37 | 3.38 |
| Bh:Bp 50:50 | 20.51 | 0.13 | 22.77 | 0.17 | 2.26 | 5.56 | 1.18 |
| Bh:Bp 25:75 | 19.83 | 0.09 | 23.15 | 0.35 | 3.32 | 6.31 | 0.44 |
| Bh:Bp 10:90 | 19.55 | 0.24 | 23.66 | 0.25 | 4.11 | 6.87 | 0.12 |
| Bh:Bp 0:100 | 20.15 | 0.10 | 24.09 | 0.07 | 3.94 | 6.75 | N/A |
| NTC | -- | -- | -- | -- | -- | -- | -- |

^a^A total of 2ng DNA was added to each PCR. Bh, *Burkholderia thailandensis-*like MSMB43; Bp, *Burkholderia pseudomallei*.

^b^ σ, standard deviation. The σ was calculated based on four replicates (see Figures S2 and 3).

^c^∆C_T_ σ>2 were considered different from pure Bh template. Using the Bh-specific probe C_T_, all mixtures were distinguishable from pure Bh template.

^d^∆C_T_ σ>2 were considered different from pure Bp template. Mixtures that were not different according to SDs are shaded.
