# Supplementary material for: Development and Validation of Burkholderia pseudomallei-Specific Real-Time PCR Assays for Clinical, Environmental or Forensic Detection Applications
Source: PLoS One. 2012 May 18;7(5):e37723. doi: 10.1371/journal.pone.0037723 (PMC3356290; doi:10.1371/journal.pone.0037723)
Supplement: Table S4 — Limit of Quantitation (LoQ) for the 122018 and 266152 assays. (DOC) [file pone.0037723.s009.doc]

| DNA | Amount of DNA (ng) | **Assay 122018 LoQ^a^** | | **Assay 266152 LoQ^a^** | |
| --- | --- | --- | --- | --- | --- |
|  |  | *B. pseudomallei* TaqMan probe | Non-*B. pseudomallei* TaqMan probe | *B. pseudomallei* TaqMan probe | Non-*B. pseudomallei* TaqMan probe |
| *B. pseudomallei* | 4x10^1^ | 8 | 0 | 8 | 8 |
| *B. pseudomallei* | 4x10^0^ | 8 | 0 | 8 | 8 |
| *B. pseudomallei* | 4x10^-1^ | 8 | 0 | 8 | 8 |
| *B. pseudomallei* | 4x10^-2^ | 8 | 0 | 8 | 8 |
| *B. pseudomallei* | 4x10^-3^ | 8 | 0 | 8 | 8 |
| *B. pseudomallei* | 4x10^-4^ | 8 | 0 | 8 | 8 |
| *B. pseudomallei* | 4x10^-5^ | 8 | 0 | 8 | 8 |
| *B. pseudomallei* | 4x10^-6^ | 1 | 0 | 8 | 8 |
| *B. pseudomallei* | 4x10^-7^ | 1 | 0 | 1 | 1 |
| *B. pseudomallei* | 4x10^-8^ | 0 | 0 | 0 | 0 |
| *B. pseudomallei* | 4x10^-9^ | 0 | 0 | 0 | 0 |
| *B. pseudomallei* | 4x10^-10^ | 0 | 0 | 0 | 0 |
| *B. pseudomallei* | 4x10^-11^ | 0 | 0 | 0 | 0 |
| *B. pseudomallei* | 4x10^-12^ | 0 | 0 | 0 | 0 |
| *B. pseudomallei* | 4x10^-13^ | 0 | 0 | 0 | 0 |
| *B. pseudomallei* | 4x10^-14^ | 0 | 0 | 0 | 0 |
| *B. thailandensis*-like | 4x10^1^ | 2 | 8 | 8 | 8 |
| *B. thailandensis*-like | 4x10^0^ | 2 | 8 | 8 | 8 |
| *B. thailandensis*-like | 4x10^-1^ | 1 | 8 | 8 | 8 |
| *B. thailandensis*-like | 4x10^-2^ | 0 | 8 | 8 | 8 |
| *B. thailandensis*-like | 4x10^-3^ | 0 | 8 | 8 | 8 |
| *B. thailandensis*-like | 4x10^-4^ | 0 | 8 | 8 | 8 |
| *B. thailandensis*-like | 4x10^-5^ | 0 | 5 | 7 | 7 |
| *B. thailandensis*-like | 4x10^-6^ | 0 | 2 | 2 | 2 |
| *B. thailandensis*-like | 4x10^-7^ | 0 | 0 | 1 | 1 |
| *B. thailandensis*-like | 4x10^-8^ | 0 | 0 | 0 | 0 |
| *B. thailandensis*-like | 4x10^-9^ | 0 | 0 | 0 | 0 |
| *B. thailandensis*-like | 4x10^-10^ | 0 | 0 | 0 | 0 |
| *B. thailandensis*-like | 4x10^-11^ | 0 | 0 | 0 | 0 |
| *B. thailandensis*-like | 4x10^-12^ | 0 | 0 | 0 | 0 |
| *B. thailandensis*-like | 4x10^-13^ | 0 | 0 | 0 | 0 |
| *B. thailandensis*-like | 4x10^-14^ | 0 | 0 | 0 | 0 |

^a^LoQ was determined as the lowest amount at which all eight replicates amplified with σ<0.8 (highlighted in orange). NB. For both assays, the upper LoQ was not reached at the highest tested amount of 40ng.
