# Supplementary material for: Development and Validation of Burkholderia pseudomallei-Specific Real-Time PCR Assays for Clinical, Environmental or Forensic Detection Applications
Source: PLoS One. 2012 May 18;7(5):e37723. doi: 10.1371/journal.pone.0037723 (PMC3356290; doi:10.1371/journal.pone.0037723)
Supplement: Table S5 — Limit of Detection (LoD) for the 122018 and 266152 assays. (DOC) [file pone.0037723.s010.doc]

| **DNA** | **Category** | **Amount of DNA (ng)** | ***B. pseudomallei* TaqMan probe** | | | **Non-*B. pseudomallei* TaqMan probe** | | |
| --- | --- | --- | --- | --- | --- | --- | --- | --- |
|  |  |  | **no. amplified** | **mean C_T_** | **SD C_T_** | **no. amplified** | **mean C_T_** | **SD C_T_** |
| **Assay 122018** | | | | | | | | |
| *B. pseudomallei* | success1 | 4x10^-4^ | 8 | 30.75 | 0.44 | --- | --- | --- |
| *B. pseudomallei* | success2 | 4x10^-5^ | 8 | 34.16 | 0.44 | --- | --- | --- |
| *B. pseudomallei* | spotty1 | 4x10^-6^ | 4 | 37.51 | 0.65 | --- | --- | --- |
| *B. pseudomallei* | spotty2 | 4x10^-7^ | 0 | und | --- | --- | --- | --- |
| *B. pseudomallei* | fail1 | 4x10^-8^ | 0 | und | --- | --- | --- | --- |
| *B. pseudomallei* | fail2 | 4x10^-9^ | 0 | und | --- | --- | --- | --- |
| *B. thailandensis*-like | success1 | 4x10^-3^ | --- | --- | --- | 8 | 32.74 | 0.34 |
| *B. thailandensis*-like | success2 | 4x10^-4^ | --- | --- | --- | 8 | 36.29 | 0.46 |
| *B. thailandensis*-like | spotty1 | 4x10^-5^ | --- | --- | --- | 2 | 39.82 | 0.23 |
| *B. thailandensis*-like | spotty2 | 4x10^-6^ | --- | --- | --- | 0 | und | --- |
| *B. thailandensis*-like | fail1 | 4x10^-7^ | --- | --- | --- | 0 | und | --- |
| *B. thailandensis*-like | fail2 | 4x10^-8^ | --- | --- | --- | 0 | und | --- |
| **Assay 266152** | | | | | | | | |
| *B. pseudomallei* | success1 | 4x10^-5^ | 8 | 34.50 | 0.48 | --- | --- | --- |
| *B. pseudomallei* | success2 | 4x10^-6^ | 6 | 37.43 | 0.81 | --- | --- | --- |
| *B. pseudomallei* | spotty1 | 4x10^-7^ | 0 | und | --- | --- | --- | --- |
| *B. pseudomallei* | spotty2 | 4x10^-7^ | 0 | und | --- | --- | --- | --- |
| *B. pseudomallei* | fail1 | 4x10^-8^ | 0 | und | --- | --- | --- | --- |
| *B. pseudomallei* | fail2 | 4x10^-9^ | 0 | und | --- | --- | --- | --- |
| *B. thailandensis*-like | success1 | 4x10^-3^ | --- | --- | --- | 8 | 31.20 | 0.60 |
| *B. thailandensis*-like | success2 | 4x10^-4^ | --- | --- | --- | 8 | 34.80 | 0.55 |
| *B. thailandensis*-like | spotty1 | 4x10^-6^ | --- | --- | --- | 4 | 38.32 | 1.07 |
| *B. thailandensis*-like | spotty2 | 4x10^-7^ | --- | --- | --- | 0 | und | --- |
| *B. thailandensis*-like | fail1 | 4x10^-8^ | --- | --- | --- | 0 | und | --- |
| *B. thailandensis*-like | fail2 | 4x10^-9^ | --- | --- | --- | 0 | und | --- |

NB. Eight replicates were tested at each data point. Green highlighted fields indicate DNA amounts that consistently give 100% amplification; orange highlighted fields denote amounts that give spotty amplification (i.e. between 1 and 7 replicates amplify); red highlighted fields constitute 100% failures. No NTCs showed detectable amplification.
