# Supplementary material for: Development and Validation of Burkholderia pseudomallei-Specific Real-Time PCR Assays for Clinical, Environmental or Forensic Detection Applications
Source: PLoS One. 2012 May 18;7(5):e37723. doi: 10.1371/journal.pone.0037723 (PMC3356290; doi:10.1371/journal.pone.0037723)
Supplement: Table S6 — Range of linearity for B. pseudomallei 122018 and 266152 TaqMan assays. (DOC) [file pone.0037723.s011.doc]

| **Assay** | **Linearity cutoffs** | ***B. pseudomallei* probe gDNA amount (ng)** | **Non-*B. pseudomallei* probe gDNA amount (ng)^c^** |
| --- | --- | --- | --- |
| 122018 | Slope, R^2^, replicate number^a^ | 4x10^-5^ to ≥40 | 4x10^-5^ to ≥40 |
|  | Slope, R^2^, replicate number, σ^b^ | 4x10^-5^ to ≥40 | 4x10^-4^ to ≥40 |
| 266152 | Slope, R^2^, replicate number^a^ | 4x10^-6^ to ≥40 | 4x10^-4^ to ≥40 |
|  | Slope, R^2^, replicate number, σ^b^ | 4x10^-6^ to ≥40 | ≥40 |

^a^Range of linearity when slope (between -3.3 and -3.7; -3.32 indicates 100% efficiency), number of amplified replicates (≥25% amplification success) and R^2^ values (> 0.997) (see Figure S4 for linear plots) are taken into account. Data points not adhering to these criteria were considered outside the linear range and were removed from analysis. In some instances, the standard deviation (σ) for replicates within this linear range exceeded a cutoff of ≥0.8. However, using eight replicates, the R^2^ values and no. of amplified replicates were acceptable; therefore, this larger range of linearity *can* be used, but it is recommended that eight replicates should be run.

^b^As above, plus σ of <0.8 taken into account.

^c^The upper limit of linearity for 122018 and 266152 was not reached; therefore, the range of linearity surpasses this amount.
