# Supplementary material for: Development and Validation of Burkholderia pseudomallei-Specific Real-Time PCR Assays for Clinical, Environmental or Forensic Detection Applications
Source: PLoS One. 2012 May 18;7(5):e37723. doi: 10.1371/journal.pone.0037723 (PMC3356290; doi:10.1371/journal.pone.0037723)
Supplement: Table S7 — Robustness summary for 122018 and 266152 TaqMan assays. (DOC) [file pone.0037723.s012.doc]

| **Temp. (^o^C)** | **DNA** | **Robustness attributes** |
| --- | --- | --- |
| 122018 ASSAY | |  |
| 57.5 | *B. pseudomallei* | Samples amplified ~1 C_T_ later than at 60^o^C |
| 60 | *B. pseudomallei* | Best temperature; most consistent amplification |
| 62.5 | *B. pseudomallei* | Highest failure rate; amplified samples had precision similar to 60^o^C |
| 57.5 | *B. thailandensis*-like | Best temperature; most consistent amplification |
| 60 | *B. thailandensis*-like | Comparable success rate to 57.5^o^C; samples amplified ~1 C_T_ later than at 57.5^o^C |
| 62.5 | *B. thailandensis*-like | High failure rate; much higher C_T_s than at 57.5 ^o^C and 60^o^C |
| 266152 ASSAY | |  |
| 57.5 | *B. pseudomallei* | Similar to 60^o^C; lower precision and samples amplified ~1 C_T_ later than at 60^o^C |
| 60 | *B. pseudomallei* | Best temperature; most consistent amplification |
| 62.5 | *B. pseudomallei* | Similar to 60^o^C; replicates amplified ~1C_T_ later than at 60^o^C |
| 57.5 | *B. thailandensis*-like | Highest failure rate; substantial loss of precision *cf*. 60^o^C |
| 60 | *B. thailandensis*-like | Best temperature; most consistent amplification |
| 62.5 | *B. thailandensis*-like | Higher failure rate *cf.* 60^o^C; much higher C_T_s than at 60^o^C |
